# Cost-effectiveness of a new autoantibody test added to Computed Tomography (CT) compared to CT surveillance alone in the diagnosis of lung cancer amongst patients with indeterminate pulmonary nodules

**DOI:** 10.1371/journal.pone.0237492

**Published:** 2020-09-02

**Authors:** Andrew John Sutton, Gurdeep S. Sagoo, Leon Jackson, Mike Fisher, Geoffrey Hamilton-Fairley, Andrea Murray, Adam Hill

**Affiliations:** 1 Institute of Health Economics, Edmonton, Alberta, Canada; 2 Test Evaluation Group, Institute of Health Sciences, University of Leeds, Leeds, United Kingdom; 3 Oncimmune Limited, City Hospital, Clinical Sciences Building, Nottingham, United Kingdom; 4 Institute of Global Health Innovation, Imperial College London, South Kensington Campus, London, United Kingdom; The Cancer Institute of New Jersey, Robert Wood Johnson Medical School, UNITED STATES

## Abstract

Oncimmune's EarlyCDT®-Lung is a simple ELISA blood test that measures seven lung cancer specific autoantibodies and is used in the assessment of malignancy risk in patients with indeterminate pulmonary nodules (IPNs). The objective of this study was to examine the cost-effectiveness of EarlyCDT-Lung in the diagnosis of lung cancer amongst patients with IPNs in addition to CT surveillance, compared to CT surveillance alone which is the current recommendation by the British Thoracic Society guidelines. A model consisting of a combination of a decision tree and Markov model was developed using the outcome measure of the quality adjusted life year (QALY). A life-time time horizon was adopted. The model was parameterized using a range of secondary sources. At £70 per test, EarlyCDT-Lung and CT surveillance was found to be cost-effective compared to CT surveillance alone with an incremental cost-effectiveness ratio (ICER) of less than £2,500 depending on the test accuracy parameters used. It was also found that EarlyCDT-Lung can be priced up to £1,177 and still be cost-effective based on cost-effectiveness acceptance threshold of £20,000 / QALY. Further research to resolve parameter uncertainty, was not found to be of value. The results here demonstrate that at £70 per test the EarlyCDT-Lung will have a positive impact on patient outcomes and coupled with CT surveillance is a cost-effective approach to the management of patients with IPNs. The conclusions drawn from this analysis are robust to realistic variation in the parameters used in the model.

## Introduction

According to Cancer Research UK (https://www.cancerresearchuk.org/health-professional/cancer-statistics/statistics-by-cancer-type/lung-cancer) there are approximately 50,000 new cases of lung cancer every year making lung cancer the third most common cancer in the UK. Furthermore, lung cancer is the most common cause of cancer death in the UK with 5- and 10-year survival as low as 10% and 5%, respectively (Cancer Research UK), which highlights the need to detect and correctly treat lung cancer as early as possible. This is particularly pertinent when imaging techniques identify indeterminate pulmonary nodules (IPNs) which are between 4mm and 20mm in size and carry a risk of malignancy of 10–70%. Often this is too low to justify a biopsy or other invasive procedure that carries a risk of morbidity and according to the British Thoracic Society Guidelines [1], an option for the clinician and patient is CT surveillance or ‘watchful waiting’. This entails repeat scanning at 3 months and 1 year to assess nodule volume doubling time (VDT). Patients with a VDT below 25% after 1 year are classed as negative and discharged, though some patients will not be discharged for up to 4 years follow up. Following the introduction of multidetector computed tomography (MDCT), the number of nodules detected, particularly those that are small, has increased dramatically with the prevalence of noncalcified lung nodules as 33% (range 17–53%) and 13% (range 2–24%), in screening and non-screening study populations, respectively [1].

Oncimmune's EarlyCDT–Lung is a simple ELISA blood test that measures seven lung cancer specific autoantibodies, and is used for the assessment of malignancy risk in patients with IPNs. Robust and easy to use, it can be run in any laboratory with standard laboratory equipment. The test has been marketed in the USA since 2012 and over 150,000 tests have been sold. A “kit” form of the test was CE marked in May 2017 for distribution to clinical laboratories outside the USA.

Using a decision analytic model, the objective of this study was to examine the cost-effectiveness of autoantibody test (AABT), EarlyCDT–Lung, in the diagnosis of lung cancer amongst patients with IPNs applied in the addition to CT surveillance, compared to CT surveillance alone as specified in the British Thoracic Society guidelines in which patients are offered surveillance through repeat CT scanning.

## Methods

### Developing the model structure

The comparison of the different testing strategies considered in this study are best represented using a modelling framework in which the various possible testing and treatment pathways can be compared. The patient group under examination in this study are 62 year old patients [2] with IPNs. Identified by imaging, these nodules are between 4mm and 20mm in size and carry a risk of malignancy of 10–65%. A model allows explicit representation of the impact of the accuracy of the tests, the costs incurred by the health care provider, and the impact on health-related quality of life (QoL) experienced by the patients that follow a particular diagnostic pathway.

A model which consists of a combination of a decision tree and Markov model was developed using TreeAge Pro 2001 software (TreeAge Software Inc., Williamstown, MA, USA). A life-time time horizon was adopted. Given that some events may occur on the patient pathways many years into the future, a Markov model approach was considered to be most appropriate.

**Fig 1:**
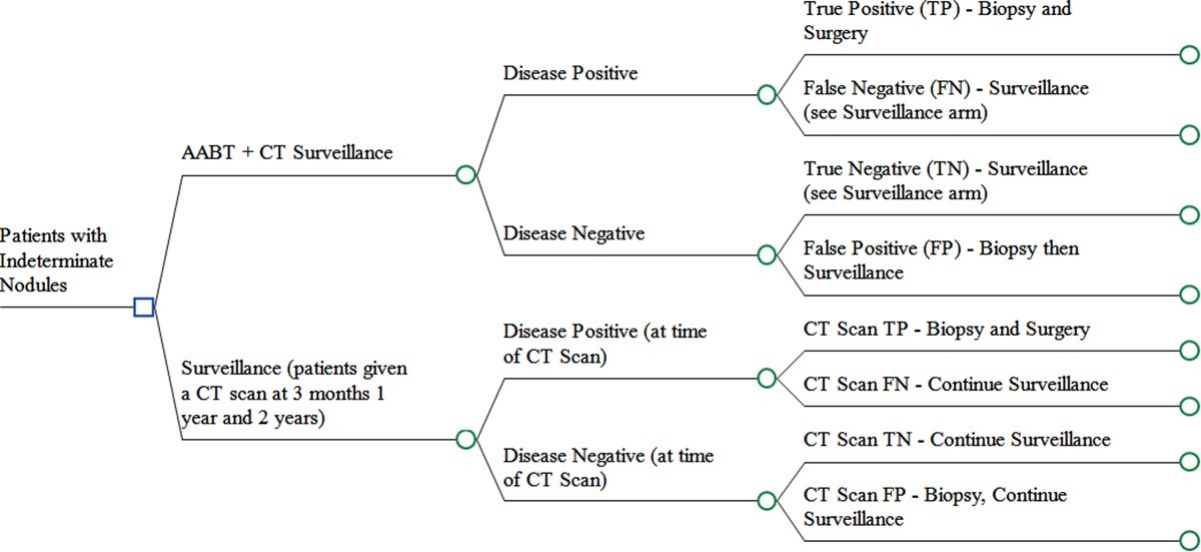
Testing pathways for the AABT and surveillance strategies.

Two different testing pathways were compared which describe alternative approaches to the testing and surveillance of these patients and their IPNs. The testing pathways are shown in Fig 1:

Fig 1 shows the testing pathways for the two strategies considered in this analysis. These are defined as follows:

- AABT+Surveillance–Patients receive the AABT (EarlyCDT-Lung), amongst those that test positive for malignancy, these are then given a biopsy followed by surgery for those that have confirmed malignancy identified through biopsy. Patients that are AABT test negative then follow the surveillance strategy below and are followed up with CT scans at 3 months, 12 months, and 24 months. Patients that are found to have a nodule that has grown at follow-up, are given a surgical biopsy. If the nodule is benign no further follow up is required; while patients that are found to have a malignant nodule receive surgery.
- Surveillance–Patients receive a CT scan at 3 months, 12 months, and 24 months. Those that test positive for malignancy following a biopsy, are then given surgery for the confirmed malignancy. Patients that test negative continue on the surveillance pathway. Again, patients that are found to have a benign nodule that has grown at follow up are given surgical biopsy.

In all cases patients that have surgery are at risk of surgery related mortality and complications.

**Fig 2:**
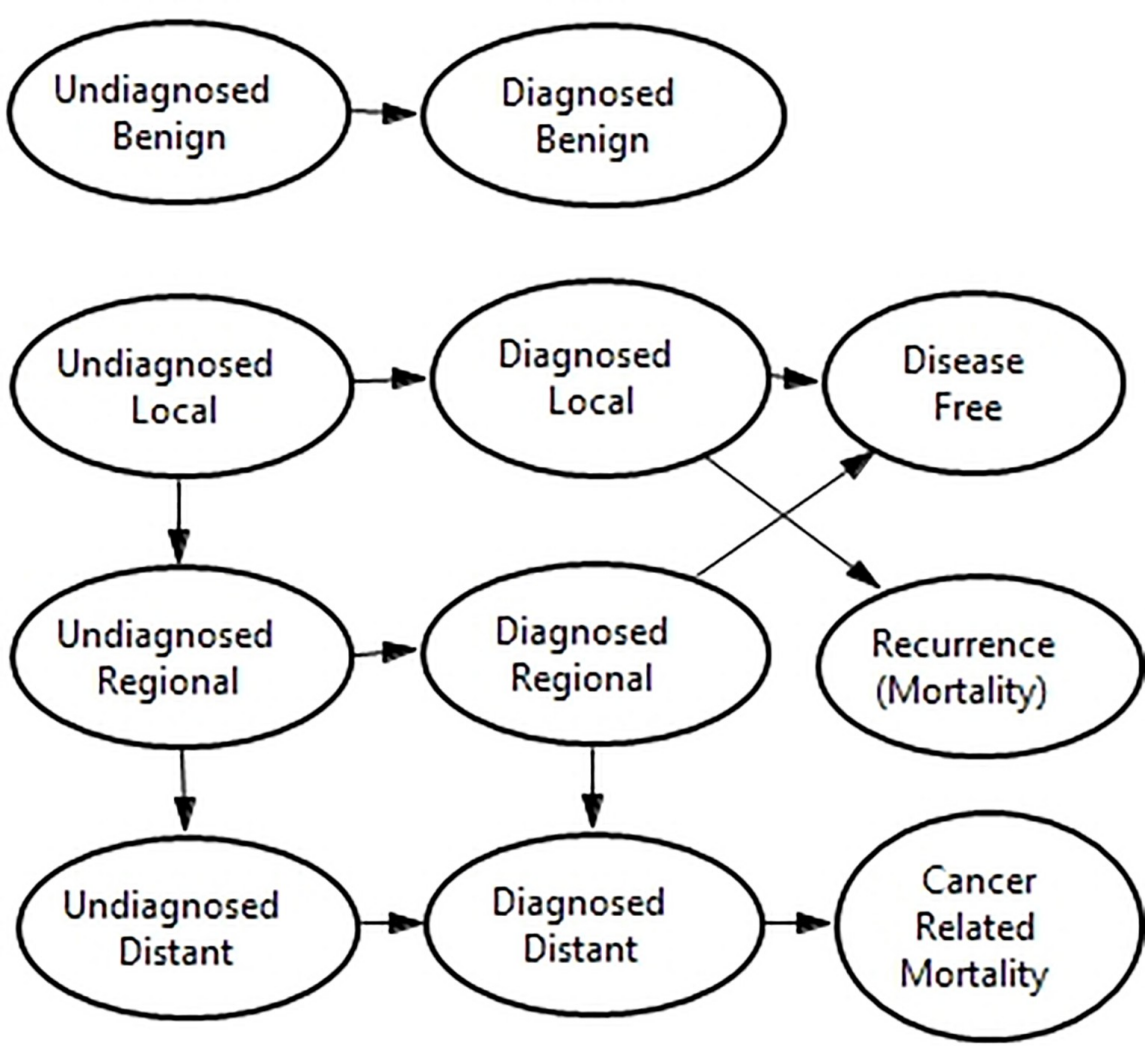
Markov model (reproduced from Gould, Sanders [2]).

Following the approach described by Gould, Sanders [2] Fig 2 shows the model structure describing the health states of patients and how these evolve over time.

Patients enter the model undiagnosed either with an undiagnosed benign nodule, or an undiagnosed malignant nodule which may be at a local, regional, or distant stage. Patients that receive a true positive test then transition to their respective diagnosed state and receive surgery if the nodule is malignant. While those that are false negative (i.e. have a malignant nodule that is missed) remain undiagnosed but may progress to a more advanced disease state in the future. Patients that transition to the diagnosed benign state remain in this state under surveillance but may require surgical biopsy in the future if their nodule is subject to significant growth. Patients in the diagnosed local and regional states are at risk of recurrence or progression respectively for a period of 5 years after which they are assumed to be disease free. Patients in the diagnosed distant states are at constant risk of cancer related mortality for the rest of their lives. In all model states, patients are also subject to all-cause mortality.

## Model assumptions

As part of the modelling framework and in order to conduct this analysis it is necessary to make some assumptions. These are described as follows:

- Biopsy is 100% accurate
- Compliance with surveillance is 100%
- Nodules are diagnosed following a CT scan after doubling/progression
- A CT scan is never performed after biopsy or surgery as imaging after an invasive diagnostic procedure is unusual [2]
- Similarly, biopsy and observation are never performed after surgery [2]
- Surgery will always be performed if biopsy confirms a malignancy [2]
- If biopsy reveals a benign diagnosis, patients are subject to surveillance [2]
- Surgical biopsy is always performed if benign nodule growth is observed during surveillance [2]
- If no growth is observed after 24 months, then nodule is assumed to be benign and no further surveillance is conducted
- CT scans performed during surveillance are 100% accurate at detecting growth in benign nodules
- Patients in the diagnosed local and regional disease states are at risk of recurrence and progression for 5 years, after which they are considered to be free from cancer
- Following a positive test, a patient is referred to a multi-disciplinary team (MDT)

## Data requirements

The data required to parameterise the economic model were obtained through the extensive use of secondary sources. The parameters used in this model can be broadly categorized into prevalence of malignancy, accuracy parameters, transition probabilities between model states, resource use and costs, and utility values.

## Prevalence

In the base case scenario, the prevalence of malignancy amongst patients presenting with IPNs was taken to be 9.5% (7/74) which is applied to both arms of the analysis. This is based on the logic as described by Edelsberg, Weycker [3] to interpret the data described in the study by Tanner, Porter [4], which is as follows:

In the study by Tanner, 74 patients were assigned to CT surveillance from an intermediate-risk group, amongst which 7 had lung cancer, yielding a lung cancer prevalence of 9.5 (7/74).

## Test accuracy

Two alternative scenarios which describe the test accuracy of the AABT test are considered in this analysis, which are based on the availability of current test accuracy parameters for the AABT. The test accuracy parameters for the tests considered in this analysis are described Table 1 below:

The data from the 12 studies used in Gould, Sanders [2] to estimate the test accuracy values for a CT scan were re-analysed using the Metandi function in Stata so that the necessary information for the probabilistic sensitivity analysis could be obtained (see below). This led to slightly different sensitivity and specificity values compared to those reported by Gould, Sanders [2] (Gould values: Sensitivity = 0.965; specificity = 0.558).

**Table 1:** Test accuracy parameter values.

Test	Sensitivity	Specificity	Reference
AABT:			
Scenario A	0.41	0.93	Taken from a ROC curve described in [5]
Scenario B	0.28	0.98	“”
CT Scan	0.923	0.723	[2] (taken from a meta-analysis of 12 studies)

## Transition probabilities

Table 2 describes the probabilities and proportions that are applied to the economic model. In all cases, unless otherwise noted, the probabilities are monthly probabilities.

**Table 2:** Transition probabilities used in the model.

*Parameter*	*Value*	*Reference*
Proportion of malignant nodules that are initially:		
Local Stage	0.875	[2]
Regional Stage	0.125 (0.078–0.165)	“”
Probability of detecting growth in a benign nodule during observation period: During first month	0.28 (0.13–0.29)	[2]
During each subsequent month	0.005 (0–0.01)	[2]
Natural Mortality	Varied by age	Office of National Statistics (2018)
Probability distant cancer related mortality		[2] (use+-50% as Gould)
0–12 months	0.1255 (+-50% Gould)	
13–24 months	0.0670 (+-50% Gould)	
25–36 months	0.0589 (+-50% Gould)	
37–48 months	0.0150 (+-50% Gould)	
Mortality Undiagnosed malignant nodule	0.02688 (+-50% Gould)	Based on a life expectancy of 36.7 months for patient with untreated 2cm nodule that doubled every 5.24 months [2]
Probability of progression from undiagnosed local to regional, and from undiagnosed regional to distant cancer	0.19224 (0.18887–0.21005; 95% CI)	[2]
Probability mortality due to biopsy	29/31960	[6]
Probability guided needle biopsy complications	230/31960	“”
Probability mortality due to recurrence		Calculated from data. See text [2]
0–12 months	0.0106	(use +-50% see Gould)
13–24 months	0.0100	
25–36 months	0.0090	
37–48 months	0.0114	
Probability mortality due regional cancer		“”
0–12 months	0.0340	(use +-50% see Gould)
13–24 months	0.0296	
25–36 months	0.0225	
37–48 months	0.0155	
Surgery related mortality for malignant nodule	0.042 (0.017–0.053)	[2]
Surgery related mortality for benign nodule	0.005 (0.002–0.016)	“”
Surgery complications for malignant nodules	0.084 (0.048–0.11)	[2]
Surgery complications for benign nodules	0.065 (0.033–0.13)	“”
Proportion of patients that receive radiotherapy with surgery	5/35	[7]
Proportion of patients that receive chemotherapy with surgery	11/35	“”

### Calculation of cancer related mortality rates

Mortality rates for patients in the local, regional, and distant cancer states post-surgery were calculated by fitting a model by maximum likelihood to data survival curves for patients with pathologically staged lung cancer (T1N0M0), pathologically staged regional lung cancer (any T N1–3 M0), and distant lung cancer (any T any N M1) from the linked Medicare claims–Surveillance, Epidemiology and End Results (SEER) tumour registry, US [2].

**Fig 3:**
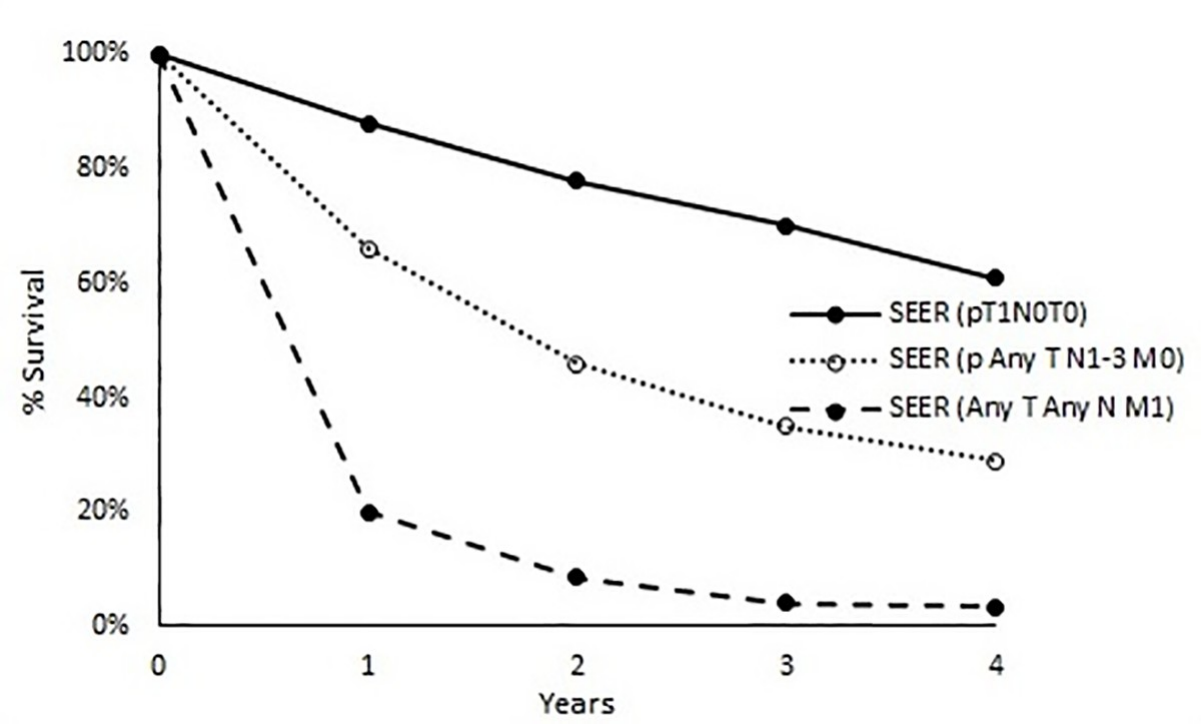
Survival curves for patients with pathologically staged local lung cancer (T1N0M0), pathologically staged regional lung cancer (any T N1-3 M0) and distant lung cancer (any T any N M1) from the linked Medicare claims–Surveillance, Epidemiology and End Results (SEER) tumour registry, USA. (Reproduced from Gould, Sanders [2]).

Local cancer-related mortality was derived from survival data for 1,207 Medicare beneficiaries with surgically treated, T1N0M0 non–small-cell lung cancer. The regional cancer-related mortality was derived from survival data for 1954 Medicare beneficiaries with pathologically staged regional lung cancer. The distant cancer-related mortality was estimated from 10 835 Medicare beneficiaries with distant-stage non-small-cell lung cancer. All data here are from the SEER tumour registry, 1990–1993, as described in Gould, Sanders [2] (Fig 3).

### Calculation of cancer progression rates

**Fig 4:**
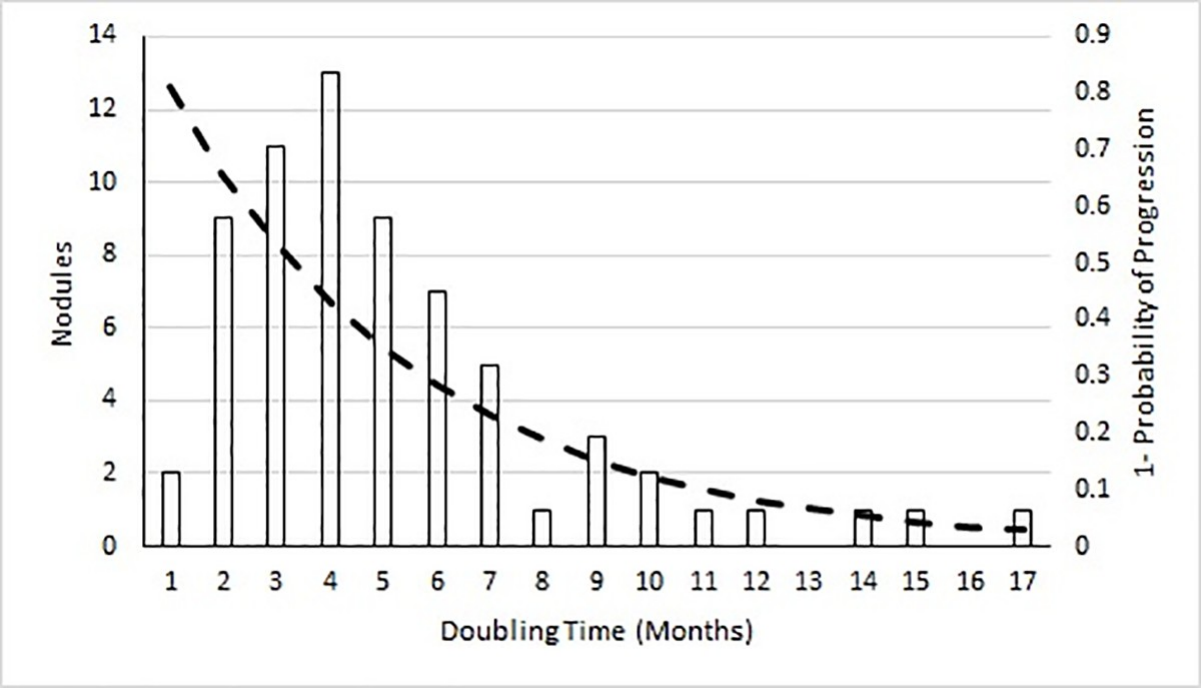
The frequency plot of the observed doubling times for 67 pulmonary nodules and mass lesions from the veterans administration–armed forces cooperative study on asymptomatic pulmonary nodules (described in Gould, Sanders [2]). Dashed line shows model output to estimate monthly progression rate.

To calculate the progression rates amongst patients with undiagnosed malignant nodules, the observed doubling times in the figure above were used. A model (probability of progression = 1-exp(-rate*t)) to obtain the monthly probability of progression was fit to the data (Fig 4) using maximum likelihood. The resulting model output showing 1 –probability of progression over time is also shown in Fig 4.

## Costs and resource use

Table 3 describes the costs applied in the economic model. All costs are in pounds (£) sterling for the 2016/17 price year. NHS Reference costs were used to attribute costs to resource use.

**Table 3:** Resource use unit costs.

*Parameter*	*Value*	*Reference (NHS Reference Costs 2016/17 unless otherwise noted)*
AABT	£70	Oncimmune
CT Scan (single area, no contrast)	£85.56	RD20A - Computerised Tomography Scan of One Area, without Contrast, 19 years and over
MDT (Multidisciplinary team)	£111.99	CMDT_Oth
Guided Needle biopsy	£948.92	DZ71Z Minor Thoracic Procedures
Surgery no complications	£7,713.03	DZ02K Complex Thoracic Procedures, 19 years and over, with CC Score 0–2
Surgery + complications	£10,177.74	DZ02H Complex Thoracic Procedures, 19 years and over, with CC Score 6+
Surgery: biopsy no complications	£3,091.08	DZ63C Major Thoracic Procedures, 19 years and over, with CC Score 0–2
Surgery: biopsy with complications	£6,733.76	DZ63A Major Thoracic Procedures, 19 years and over, with CC Score 6+
Radiotherapy	£3,252	[7], inflated from 2011/12 prices (£3,039)
Chemotherapy	£4,155.15	[7], inflated from 2011/12 prices (£3,883)

## Utility values

The utility values used in the analysis to inform the quality adjusted life year (QALY) are described in Table 4 below.

**Table 4:** Utility values.

*Parameter*	*Value*	*Reference*
Age 55–64	0.810	[8] EQ-5D index value population norms for the UK–England, using country specific Time-trade off (TTO) values
Age 65–74	0.773	“”
Age 75+	0.703	“”
Serious adverse event due to biopsy	-0.2	[6]
Local	0.71	n = 33 (stage IA and IB) at 12 months [9]
Regional	0.65	n = 12 (stage I and Stage II) at 12 months “”
Distant	0.62	n = 4 (stage IV) at 12 months “”

## Analysis

This model-based economic evaluation utilizes the primary outcome of the cost per QALY, where one QALY is defined as one year lived in perfect health. A time step of 1 month was applied in the Markov model with a life-time time horizon. This time horizon was chosen to allow the full impact of the interventions that may occur many years in the future to be included. Half cycle correction was incorporated into the analysis. Discounting was applied at 3.5% for costs and outcomes as recommended by NICE [10], with the analysis conducted from the health-care provider perspective The results are presented using the incremental cost-effectiveness ratio (ICER) which is defined as the difference in the costs of the two strategies divided by the difference in their outcomes, and net-monetary benefit (NMB) which is defined for each strategy as:

NMB=QALYsgainedxwillingnesstopay(WTP)foraQALY–Costoftheintervention.

Where the WTP for the QALY is taken to be £20,000, which is at the lower end of the £20,000 to £30,000 acceptance threshold as recommended by NICE [10].

## Sensitivity analysis

This analysis contains a number of important uncertainties that must be examined. These were examined through one-way and probabilistic sensitivity analyses.

Given this is an early economic evaluation the optimum price of the AABT test is examined. The price of the AABT test was varied to show the point at which the price of the test leads to an ICER of £20,000/QALY which is at the low end of the threshold for acceptance of an intervention as given by NICE [10].

Probabilistic sensitivity analysis (PSA) was implemented by using Beta distributions where data made this possible, using the method of moments to obtain the Alpha and Beta parameters in each case [11]. Where a range was described for parameter uncertainty, then the standard error for the Beta distribution was estimated as follows:

SE=(U‐L)/(2×1.96)

Where U and L are the upper and lower limits of the range respectively [12].

In order to apply probabilistic sensitivity analysis (PSA) to the CT scan test accuracy values the following equation was used

$$logit(sensitivity)={\Lambda e}^{-\frac{\beta }{2}}-e^{-\beta }logit(specificity)$$

From the metandi output in Stata (see Test Accuracy Section above), specificity was 0.7234 (se = 0.0276), Λ was found to be 3.156 (se = 0.2296) and β = -0.5362433. The resulting sensitivity was estimated by sampling from a beta distribution for the specificity and sampling from a normal distribution for Λ. Beta was kept constant.

## Expected value of perfect information

The expected value of perfect information (EVPI) is based on the probability of a decision maker making the wrong decision about which testing strategy to choose and the impact of making that wrong decision. It provides insights into the maximum value of conducting further research to resolve the uncertainty in the parameter values. Expected value of perfect parameter information (EVPPI) takes this idea forward and shows the maximum value of resolving the uncertainty in specific parameters or specific groups of parameters. Both methods are useful in providing insights into the direction of future research. The methods used here to estimate the EVPI and EVPPI are those described by Strong, Oakley [13].

## Results

The results here are presented for two scenarios based on alternative estimates for the test accuracy of the AABT test (Scenario A–Sensitivity 0.41 Specificity 0.93; Scenario B–Sensitivity 0.28 Specificity 0.98).

### Scenario A

At a price of £70, the cost-effectiveness results for AABT vs Surveillance for Scenario A showing the average cost and QALYs gained per patient are shown in Table 5 below:

It can be seen that when the price for AABT = £70, and adopting the test accuracy parameters as described for Scenario A, AABT+Surveillance is more costly and more effective in terms of QALYs gained than surveillance alone. Given that the ICER is well under £20,000, AABT+Surveillance can certainly be regarded as cost-effective.

**Table 5:** Cost-effectiveness of AABT vs. surveillance for testing Scenario A, where the price of AABT = £70.

Scenario A:					
	*Total Cost*	*Inc*. *Cost*	*QALYs Gained*	*Inc*. *QALYs*	*ICER (Cost/QALY)*
Surveillance	£2,261		10.6850		
AABT+Surveillance	£2,410	£149	10.7465	0.0614	£2,417

**Fig 5:**
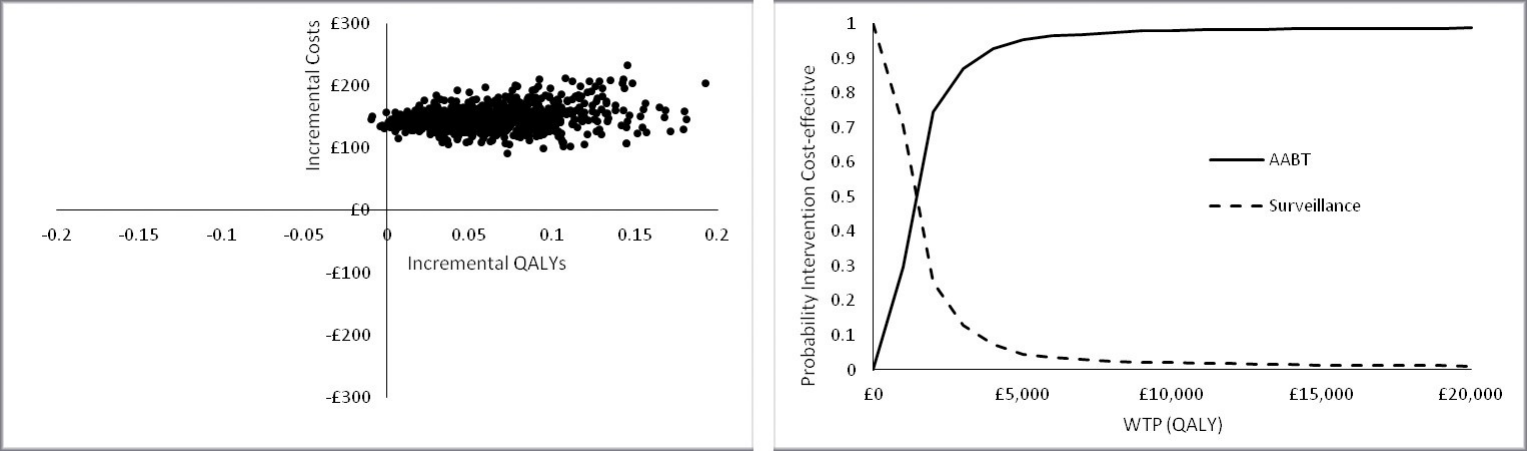
Probabilistic sensitivity analysis results for Scenario A for 1,000 model runs showing the cost-effectiveness plane and the cost-effectiveness acceptability curve for Scenario A.

The results when the uncertainty in the parameter values is considered in the analysis for Scenario A are shown in Fig 5 above. It can be seen that AABT+Surveillance is always more costly than surveillance alone and almost always (99.4%) more effective in terms of QALYs gained. The cost-effectiveness acceptability curve (CEAC) shows that AABT+Surveillance is more likely to be cost-effective at a WTP for the QALY of £2,000 and above. At a WTP of £20,000/QALY AABT is approximately 99% likely to be cost-effective.

**Fig 6:**
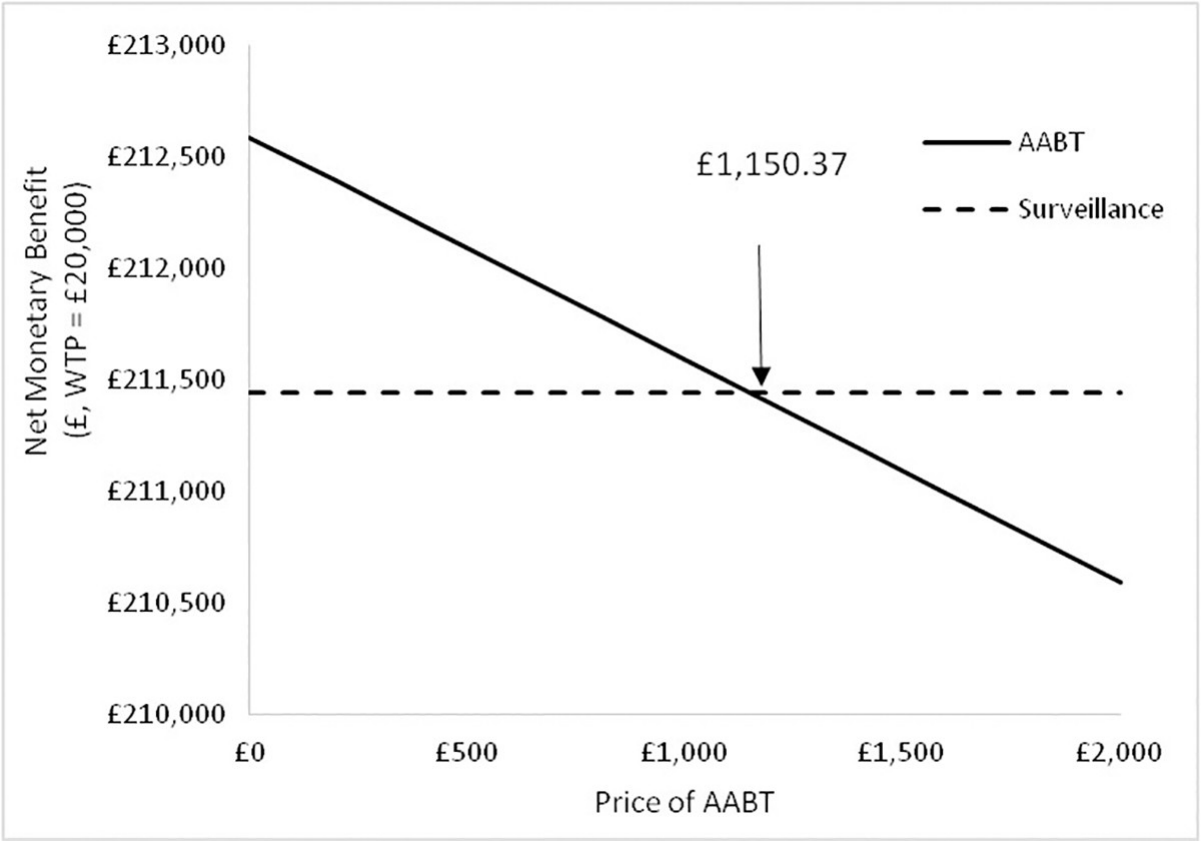
Net monetary benefit at a WTP = £20,000 / QALY for the AABT+Surveillance and surveillance strategies with variation in the price of the AABT test for Scenario A.

The net benefit for the AABT+Surveillance and Surveillance alone scenarios with variation in the price of the AABT test for Scenario A are shown in Fig 6.

It can be seen that the price of the AABT can be up to £1,150.37 and still have greater NMB than compared to Surveillance alone (WTP = £20,000). Above this price, surveillance alone becomes more cost-effective.

Assuming an annual incidence of 50,000 patients presenting with IPNs, a discount rate of 3.5% and a 10-year time horizon after which this technology will be superseded, the expected value of information for Scenario A is shown in the figure below.

**Fig 7:**
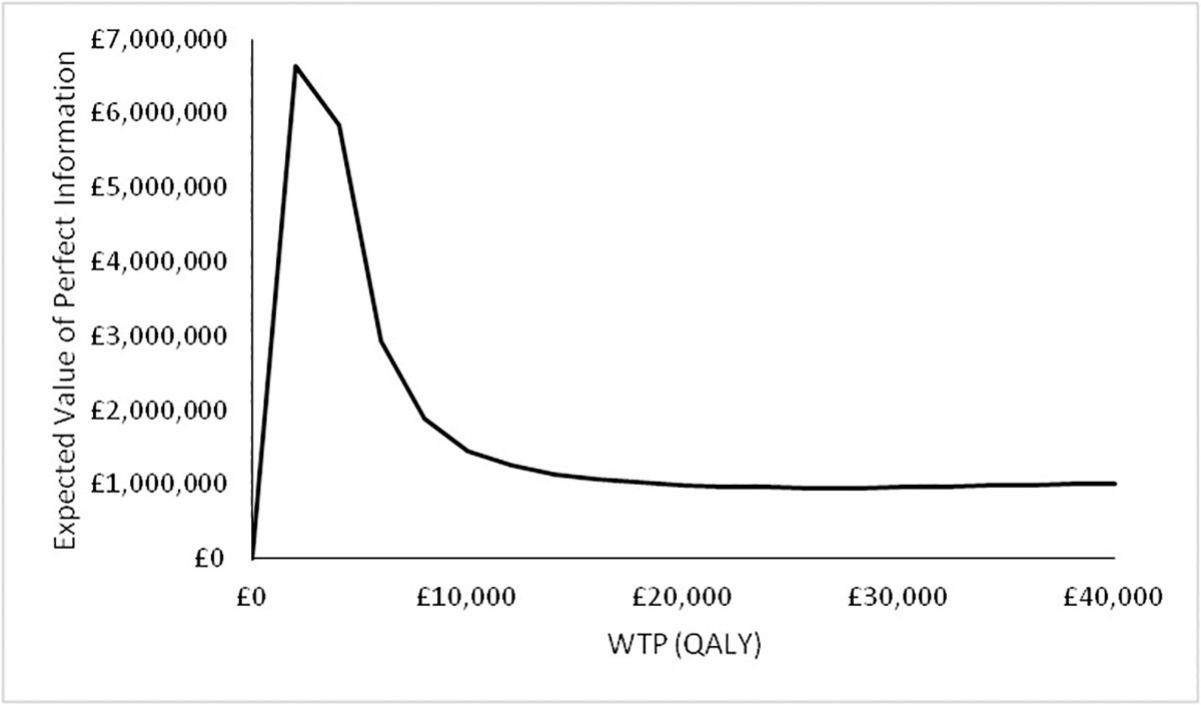
Expected value of information for Scenario A. Assuming an incidence of 50,000 new patients presenting with IPNs, a discount rate of 3.5% and a 10-year time horizon.

It can be seen in Fig 7 that at a WTP for the QALY of £20,000, the EVPI for Scenario A is approximately £1,000,000.

**Fig 8:**
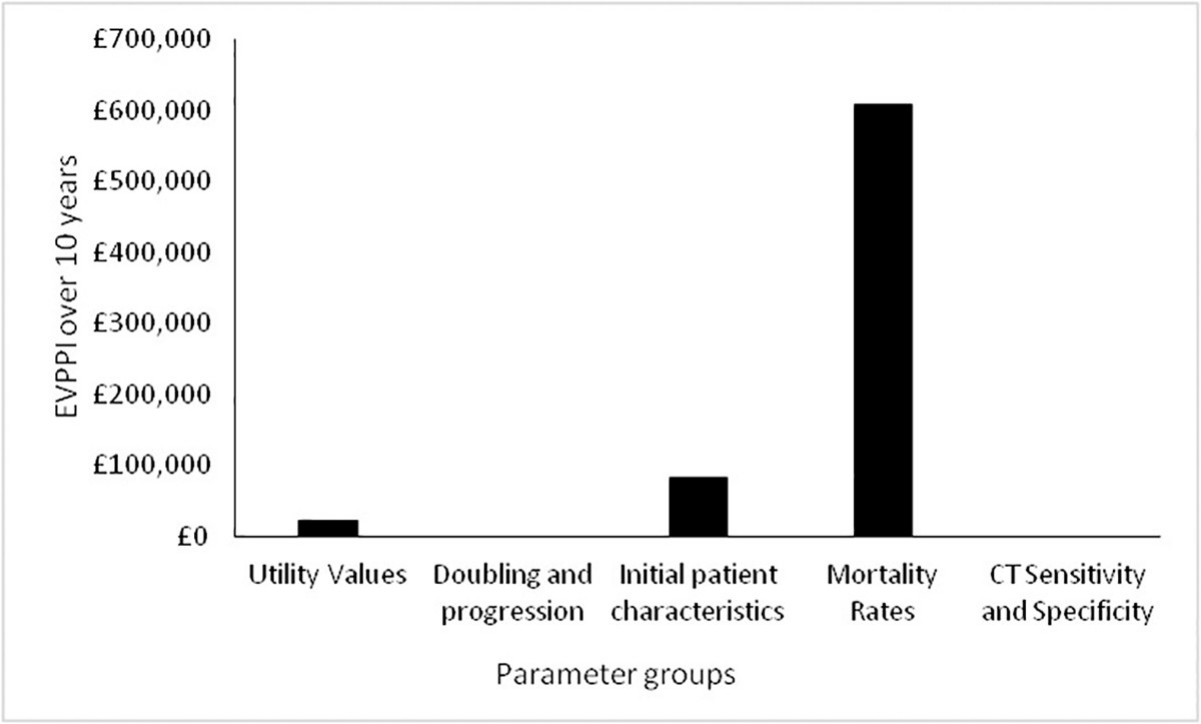
Expected value of perfect parameter information for Scenario A.

In terms of expected value of perfect parameter information. The EVPPI for different groups of parameters for Scenario A are shown in Fig 8 below.

It can be seen (Fig 8) that there is some value in resolving the uncertainty in the disease related mortality rates, initial patient characteristics (i.e. prevalence and proportion of patients with local and regional disease) and the utility values. The most value of resolving this uncertainty over 10 years is achieved by targeting the mortality rates (approximately £600,000), while the values for the other parameters is actually very low being approximately £80,000 for the initial patient characteristics and £25,000 for the utility values.

### Scenario B

Similar to Scenario A, when the price for AABT = £70, and adopting the test accuracy parameters as described for Scenario B, AABT+Surveillance is more costly and more effective in terms of QALYs gained than Surveillance alone. Again, given the low ICER value, AABT can certainly be regarded as cost-effective (Table 6).

**Table 6:** Cost-effectiveness of AABT+Surveillance vs. surveillance alone for testing Scenario B, where the price of AABT = £70.

Scenario B:					
	*Total Cost*	*Inc*. *Cost*	*QALYs Gained*	*Inc*. *QALYs*	*ICER (Cost/QALY)*
Surveillance	£2,261		10.6850		
AABT+Surveillance	£2,358	£97	10.7308	0.0457	£2,121

**Fig 9:**
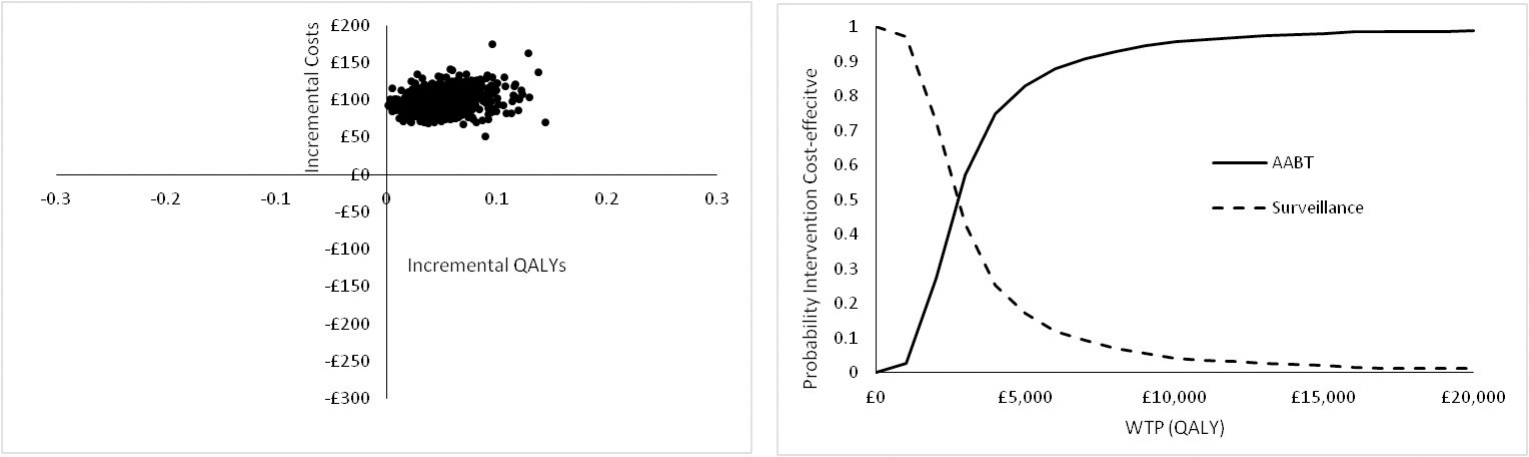
Probabilistic sensitivity analysis results for Scenario B for 1,000 model runs showing the cost-effectiveness plane and the cost-effectiveness acceptability curve for Scenario B.

It can be seen from the results of the PSA for Scenario B (Fig 9) that AABT+Surveillance is always more costly than surveillance alone and always more effective in terms of QALYs gained. The cost-effectiveness acceptability curve (CEAC) shows that AABT+Surveillance is more likely to be cost-effective at a WTP for the QALY of £3,000 and above. At a WTP of £20,000/QALY AABT is more than 98% likely to be cost-effective.

**Fig 10:**
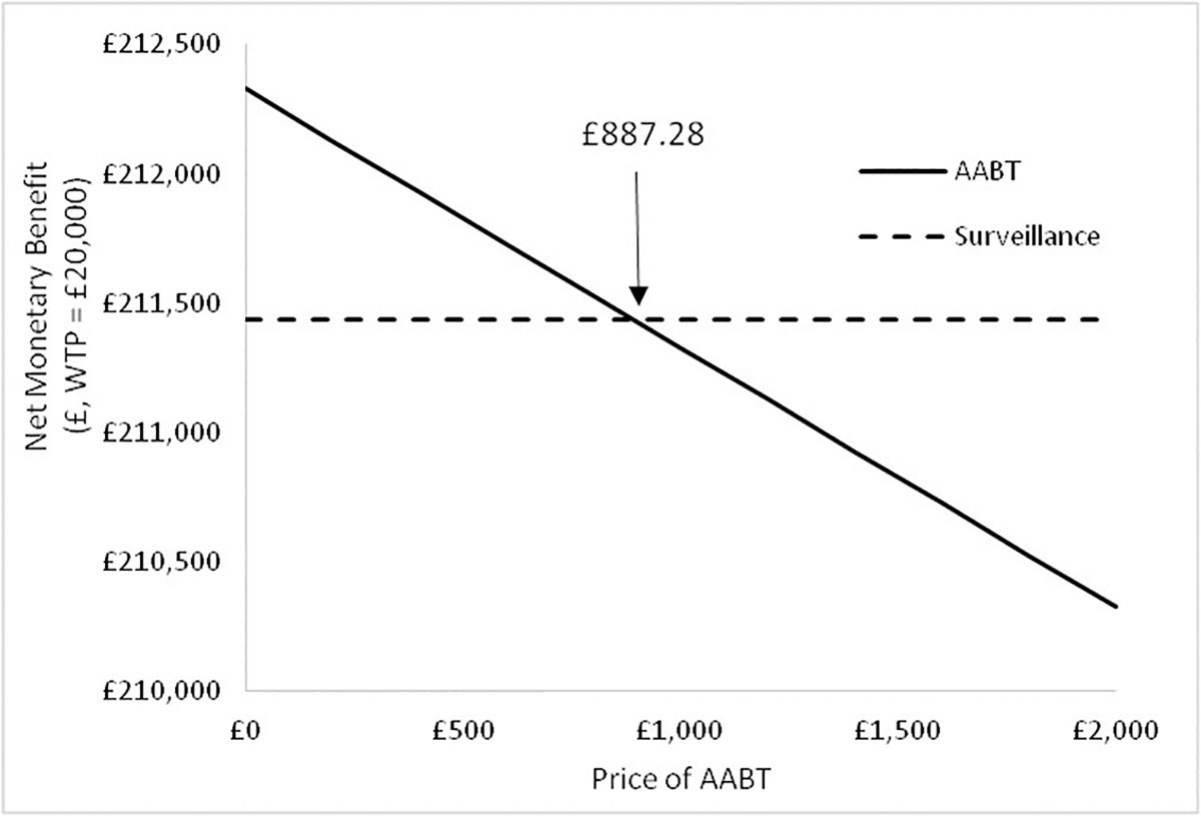
Net monetary benefit at a WTP = £20,000 / QALY for the AABT+Surveillance and surveillance alone strategies with variation in the price of the AABT test for Scenario B.

The net benefit for the AABT+Surveillance and Surveillance scenarios with variation in the price of the AABT test for Scenario B are shown in Fig 10.

In the case of the test accuracy parameters in scenario B the AABT test can be priced up to £887.28, and be more cost-effective than surveillance alone (WTP = £20,000/QALY).

**Fig 11:**
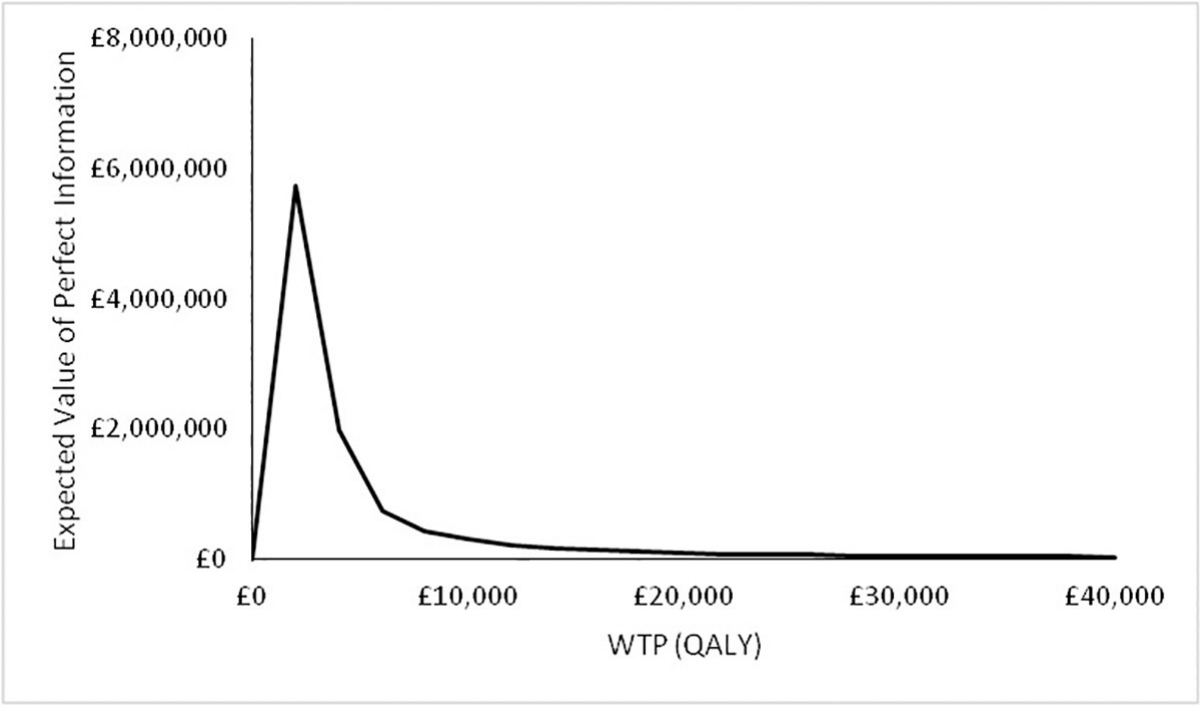
Expected value of information for Scenario B. Assuming an incidence of 50,000 new patients presenting with IPNs, a discount rate of 3.5% and a 10-year time horizon.

It can be seen in Fig 11 that at a WTP for the QALY of £20,000 the EVPI for Scenario B is approximately £100,000. In the case of Scenario B, there was found to be no value in resolving the uncertainty in any of the parameter groups (not shown).

### Analysis of Scenario A and Scenario B

Given that both Scenarios A and B show that AABT+Surveillance is cost effective compared to surveillance alone, it is important to establish whether the extra QALYs gained from Scenario A compared to Scenario B are worth paying for.

As shown in Table 7, the ICER for AABT+Surveillance Scenario A compared to Scenario B is £3,277 which is well below the NICE acceptance threshold of £20,000. Thus, it can be concluded that Scenario A has the most cost-effective test accuracy parameters and as such these should be adopted.

**Table 7:** Cost-effectiveness results showing a comparison between Surveillance and AABT for Scenarios A and B.

	*Total Cost*	*Inc*. *Cost*	*QALYs Gained*	*Inc*. *QALYs*	*ICER (Cost/QALY)*
Surveillance	£2,261		10.6850		
AABT+Surveillance Scenario B	£2,358	£97	10.7308	0.0457	£2,121.43
AABT+Surveillance Scenario A	£2,410	£52	10.7465	0.0157	£3,277.41

**Fig 12:**
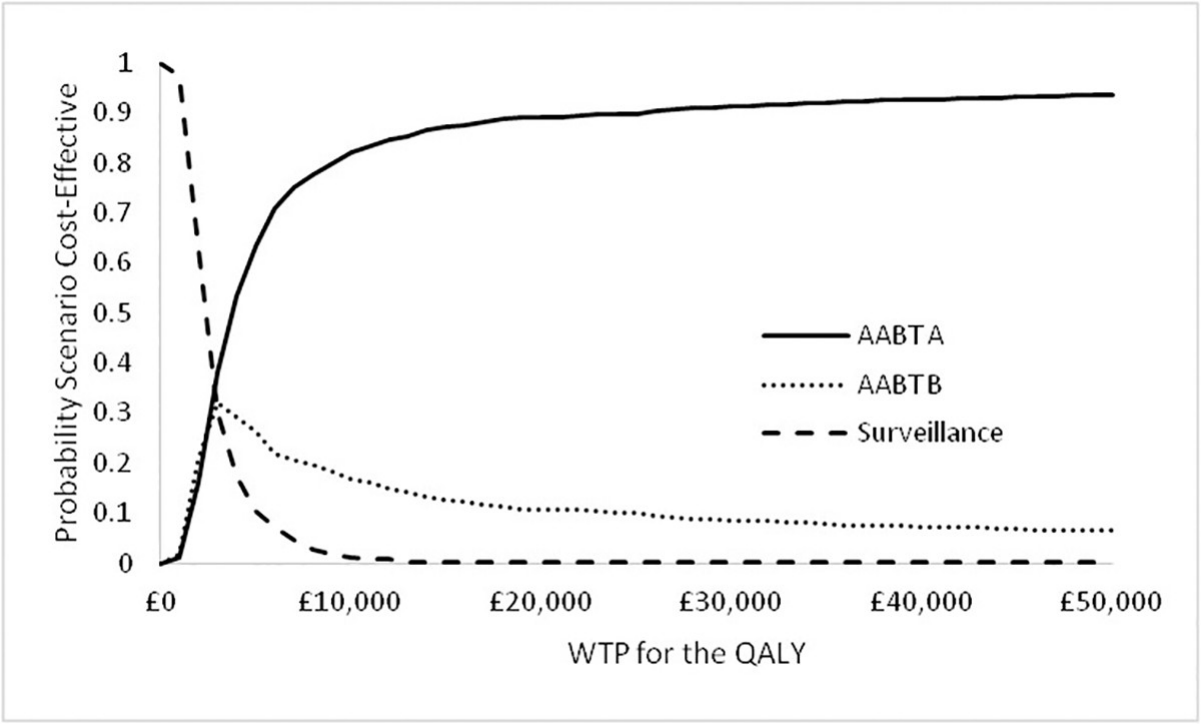
Cost-effectiveness acceptability curve comparing surveillance, AABT+Surveillance Scenario A, and AABT+Surveillance Scenario B.

The CEAC for the 3 scenarios is shown below:

It can be seen from the CEAC shown in Fig 12 that up to a WTP for a QALY of approximately £2,000, Surveillance alone is most likely to be the most cost-effective scenario, and then from WTP of approximately £3,000 upwards AABT+Surveillance Scenario A is most likely to be cost-effective. At a WTP for a QALY of £20,000 Scenario A is approximately 90% likely to be the most cost-effective option, with this probability increasing with increased WTP values.

## Discussion

Using a decision modelling framework consisting of a decision tree and Markov model the analysis here has examined the cost-effectiveness of the AABT test in addition to CT surveillance compared to the current practice of CT surveillance alone for patients with indeterminate pulmonary nodules (IPNs), as recommended in the British Thoracic Society guidelines. Two alternative pairs of test accuracy parameters were considered for the AABT test. Scenario A (sensitivity 41% specificity 93%) with its higher sensitivity and lower specificity compared to Scenario B (sensitivity 28% Specificity 98%). This analysis took a baseline price of the AABT test to be £70, but also investigated the maximum price the test could be set at, and still remain cost-effective at these values.

Based on the results of this analysis at baseline, it is quite clear that the use of the AABT test in addition to CT surveillance is cost-effective compared to CT surveillance alone. And while at £70 per test, the test was never found to be cost saving, based on the NICE threshold of acceptance (i.e. less than £20,000 / QALY) the extra effect in terms of QALYs was always found to be worth paying for. In terms of which test accuracy parameters should be adopted, again the results here are clear, with Scenario A (Sensitivity 41% Specificity 93%) being the preferred option. The probabilistic sensitivity analysis supports the main conclusions and indeed provide reassurance that these results are robust to realistic variations in the input parameters. Thus, it can be concluded that the extra sensitivity of Scenario A compared to Scenario B (41% vs. 28%) at the expense of some specificity (93% vs. 98%) leads to improved patient outcomes that are worth paying for.

The results here also demonstrate that at £70, the AABT test is significantly under-priced and could be priced at between approximately £900 to £1,170 (depending on the Scenario) and still be cost-effective based on the NICE acceptance threshold for the QALY. Although it is acknowledged that while this would still be under the NICE acceptance threshold, the increased budget impact would obviously make this much less attractive to decision makers.

“The intuition behind the results here is that by adding the additional AABT diagnostic test to current surveillance, there is a positive trade off between the patient benefits associated with the detection of true positives and the negative impact of a slight increase in the number of false positive test results due to imperfect specificity that will lead to a further biopsy. However, given the very high specificity of the AABT test **and the relatively high prevalence of malignancy (approx. 10%)**, there is very little downside to a patient receiving this test, apart from the cost of the test. If a patient is found to require surgery as a result of a true positive test, then this clearly has a positive impact on patient outcomes, while a false negative test simply leads the patient to the surveillance test pathway, which is what the patient would have received in the absence of the AABT test. Even though it could be argued that the sensitivity is still relatively low (41% is the highest in Scenario A), this is still sufficient to have a positive impact on patient outcomes.”

The conclusions drawn from this analysis are supported in the study by Edelsberg, Weycker [3] which also found the AABT test in addition to CT surveillance to be cost-effective compared to surveillance alone. However, our analysis differs from the approach taken by these authors in that we use a Markov model which allows for patients to be followed over their life-time. While Edelsberg, Weycker [3] do attempt to draw conclusions over longer time horizons, the Markov model is regarded as the most appropriate model design for chronic diseases.

This analysis has a number of limitations that have to be acknowledged. The cost of palliative care for patients that die of lung cancer have not been incorporated into this analysis. However, given that patient outcomes are improved in the AABT plus surveillance scenario, their inclusion would cause the AABT to appear even more cost-effective than has been presented in these results. Rather than doing an extensive systematic review to identify the best available evidence to populate the model, this study has made extensive use of the parameters, data and model structure from the study by [2]. While this should be regarded as a limitation, the uncertainty in the parameter values used has have been subject to extensive sensitivity analysis and this has shown that the conclusions drawn from this analysis are robust to realistic variation in the parameter values.

## Conclusion

The results here have demonstrated that the use of the EarlyCDT–Lung AABT test in addition to CT surveillance will have a positive impact on patient outcomes and is a cost-effective approach to the management of patients with IPNs, with all the results well under the NICE threshold for acceptance. The conclusions drawn from this analysis are also very robust to realistic variation in the parameters used in this model.
